# Effects of physical fitness training on the mental and physical health of stroke survivors

**DOI:** 10.12968/bjnn.2023.19.Sup2.S16

**Published:** 2023-05-03

**Authors:** V. Benedetto, D.L. Christian, A.S.R. McLoughlin, E. Smith, C. Miller, J. Hill, C.E. Lightbody, C.L. Watkins

**Affiliations:** 1 Applied Health Research hub, University of Central Lancashire (UCLan), Preston, UK; 2 Methodological Innovation, Development, Adaptation and Support (MIDAS) Theme, NIHR Applied Research Collaboration North West Coast (ARC NWC); 3 IMPlementation and Capacity building Team (IMPaCT), NIHR ARC NWC; 4 School of Nursing, UCLan, Preston, UK; 5 Lancashire Teaching Hospitals NHS Foundation Trust

**Keywords:** Stroke, Physical fitness training, Cardiorespiratory training, Resistance training, Mixed training, Rehabilitation

## Abstract

After a stroke, physical activity can be key in enhancing the rehabilitation of patients and preventing a secondary stroke. In this commentary, we critically appraise a systematic review which investigated how different types of physical fitness training impact on the mental and physical conditions of stroke survivors. Cardiorespiratory, resistance and mixed training (especially when including walking) can improve key outcomes such as the balance and mobility of stroke survivors, but the most suitable type of training depends on the individual needs and aims of the rehabilitation process. More research is needed to understand how the effects of the different types of training vary by considering the time between stroke and intervention onset, stroke severity, and the dose of intervention.

## Introduction

Physical activity is recognised as being crucial to prevent the development of several health conditions as well as generally being good for mental and physical health (Parliamentary Under Secretary of State for Public Health and Primary Care 2019). Physical activity after stroke can reduce disability, aid rehabilitation of the physical effects, and prevent further events (Saunders et al. 2016; Turan et al. 2017). However, the benefits of different types of physical activity after stroke are not well understood, which may be preventing the integration of physical activity in stroke rehabilitation and secondary stroke prevention (Fini et al. 2021). The systematic review by Saunders et al. (2020) (Saunders et al. 2020), which updates the earlier 2016 review (Saunders et al. 2016), aimed to review the evidence that examines whether specific types of physical fitness training are beneficial for health and function in people who have had a stroke, and to determine the effects of training on multiple outcome measures.

## Aim of commentary

This commentary aims to critically appraise the methods used within the review by Saunders et al. (2020) (Saunders et al. 2020) and expand upon the findings in the context of clinical practice.

## Methods

In the review by Saunders et al. (2020) (Saunders et al. 2020), a comprehensive multi-database search was implemented covering the period up to July 2018. Supplementary searches on trials’ and theses’ registries, for grey literature and forward citation searching were undertaken.

In the study selection, the authors included randomised controlled trials (RCTs) which assessed the effects of cardiorespiratory, resistance, or mixed training on adult stroke survivors. Studies were excluded if they focused on fitness training based on virtual reality approaches or combined with assistive technologies and the comparison between upper and lower body training without considering a non-exercise control group.

Two reviewers independently screened the titles and abstracts of all the retrieved studies, and the full text of those potentially eligible for inclusion. One reviewer performed the data extraction of the included studies with another reviewer validating data entry. Two reviewers independently performed a risk of bias assessment of the included studies using the Cochrane Risk of Bias tool (Higgins and Altman 2017).

Random effects meta-analyses were carried out when studies were considered sufficiently similar, reporting appropriate measures of effects and 95% CIs at the end of intervention (i.e. training period) and at the end of follow-up. Heterogeneity across the studies was reported using the I^2^ statistic as part of forest plots; where I^2^ was higher than 50% (indicating substantial heterogeneity) further exploration of the causes of variation, also through subgroup and sensitivity analyses, was attempted. Reporting bias was investigated using a funnel plot when meta-analyses were based on at least 10 studies. The quality of the evidence underpinning the different outcomes was assessed using GRADE.

## Results

In the review by Saunders et al. (2020) (Saunders et al. 2020), 17 studies were added to the 58 already examined by the previous version (Saunders et al. 2016). Therefore, the final analysis included a total of 75 studies (involving 3617 participants).

For the effects of the different types of training at the end of the intervention the authors reported the following results. For cardiorespiratory training, the meta-analysis showed moderate clinical improvements to disability levels (supported by moderate-certainty evidence, according to GRADE criteria). Furthermore, there was a clinically and statistically significant improvement on physical fitness in terms of VO_2_ peak (moderate-certainty evidence) and on mobility in terms of gait speed and gait endurance (both supported by high-certainty evidence). Physical function measured by the Berg Balance Scale was also improved but the clinical effect was small (moderate-certainty evidence), while clinically and statistically significant effects were found for the 3-metre Timed Up and Go measure (moderate-certainty evidence). No evidence of difference was found for the effects on death by cardiorespiratory training (low-certainty evidence), while not enough data was available to analyse the effects on death or dependence. However, the rates of mortality were very low within both groups.

Resistance training had positive effects on three physical fitness outcomes specifically related to muscle strength, as measured by a composite measure (moderate effect, low-certainty evidence), paretic knee flexion (moderate effect, low-certainty evidence), and paretic knee extension (large effect, low-certainty evidence). Clinically and statistically significant results were also found for resistance training on mobility outcomes related to gait speed (moderate-certainty evidence) and gait endurance (moderate-certainty evidence). On the contrary, the authors did not find statistically significant effects for resistance training on physical function in terms of the 3-metre Timed Up and Go measure (low-certainty evidence), while statistically significant but clinically modest effects were identified for the Berg Balance Scale (low-certainty evidence). The results on the effects on death by resistance training did not identify evidence of difference (low-certainty evidence), while the effects on death or dependence were not explored due to lack of data.

Mixed training showed small positive effects on disability levels (low-certainty evidence). Small positive effects were also found for physical fitness but only in terms of muscle strength, as measured by knee extension (low-certainty evidence). Other effects on physical fitness were not statistically significant, as measured by VO_2_ peak and muscle strength (specifically, ankle dorsiflexion and paretic grip strength). The results for mobility captured clinically and significantly effects for mixed training on gait speed (moderate-certainty evidence), and on gait endurance (low-certainty evidence). Statistically significant but clinically small effects were found for physical function in terms of the Berg Balance Scale (low-certainty evidence), while for the 3-metre Timed Up and Go measure the results were not statistically significant. Similarly to the other types of training, the authors found no evidence of difference by mixed training on death (low-certainty evidence), and the lack of data did not allow an analysis of the effects on death or dependence.

Sub-group analysis was conducted to directly compare the different types of training on disability outcomes at the end of intervention, with cardiorespiratory training showing moderate effects (with substantial heterogeneity) and mixed training showing small effects (with low heterogeneity), while no evidence of difference was found for respiratory training.

Due to lack of data, inconclusive effects were found on other outcomes such as cardiometabolic risk factors, quality of life, mood, and cognitive function. No statistically significant results were found for the other timepoint considered besides the end of the intervention (i.e., end of follow-up).

## Commentary

### Critical appraisal

We used the AMSTAR2 to critically appraise the quality of the review (Shea et al. 2017). While we believed that the review was of good quality for half of the AMSTAR2 domains (i.e., 8 out of 16 domains), the review seems to fall short of adequate quality in the other domains connected with both the Methods and Results sections. First, the authors do not seem to provide a justification behind the restrictions used in the eligibility criteria, for example on the exclusion of non-RCTs studies from the review and the exclusion of studies not written in English. Second, within the description of the included studies, while the authors appropriately report the vast majority of the studies’ characteristics, the indication (if any) of the different timeframes for follow-up seems missing. Third, the technique adopted to weigh the studies in the random effects meta-analyses (for example, whether the DerSimonian and Laird method was employed) is not reported. Fourth, in the interpretation of the results the likely impact on the results of the studies’ risk of bias and heterogeneity is not amply discussed. Lastly, the authors did not report the sources of funding for the studies included in the review, nor did they report the presence or absence of their individual conflicts of interest and funding sources.

### Implications for practice

Due to the heterogeneity of outcomes assessed in the included studies in the review, it is difficult to indicate which intervention type is better or worse for a specific outcome. Where common outcomes were assessed, such as disability and mobility, on visual inspection there was not a clear statistically significant difference between cardiovascular, resistance, and mixed training. Therefore, for these outcomes all three methods may provide similar levels of improvement. However, for specific outcomes relating to the purpose of the training, such as cardiovascular exercise and VO2 peak, there was a clear clinically significant improvement. Similarly, with resistance training there was a notable clinically significant improvement in strength. However, these outcomes were not assessed in the alternative exercise type and subsequently cannot be compared. Therefore, when there is a specific focus on improving physical fitness or strength the corresponding exercise type should be selected.

All three types of training (cardiovascular, resistance, and mixed training) produced an improvement in balance, with resistance training demonstrating the greatest, but not statistically significant, improvement. Therefore, when deciding on methods for improving balance other factors may be considered such as the environment, patient preference, clinician experience, equipment, and functional purpose (Banks et al. 2012; Débora Pacheco et al. 2021; Geidl et al. 2018; Simpson et al. 2011). Regarding the safety of these types of exercise, there were very low mortality rates which suggest that these exercises are safe for this population. However, the study population did include predominantly younger stroke patients (mean age approximately 62).

### Recommendations for future research

A wide range of variability in study population and intervention type was reported across studies. Further research would benefit from exploring the impact of cardiorespiratory fitness, resistance, and mixed training dependent on time between stroke and intervention onset, stroke severity, and effects of the dose of intervention. Studies with longer follow-up data are also recommended in order to determine whether and how the benefits of fitness training persist over time.

Moreover, we suggest the involvement of patient groups to co-design appropriate and acceptable attention control in future studies. Along similar lines, a better understanding of the effects on patients can be enhanced by using Patient-Reported Experience (PREMs) and Outcome Measures (PROMs).

Lastly, this review did not assess the evidence around the cost-effectiveness of the different interventions. As such, the economic evidence base will need to be scrutinised in future reviews to determine, and possibly rank, the interventions in terms of overall costs, resource use, and cost-effectiveness.

### CPD reflective questions

1) What do you think about the choice of primary and secondary outcomes that the authors decided to investigate?
2) In your opinion, what is the main take-away message from this review and why?
3) IIf you were asked to update this review, would you apply any changes to its design (for example, the types of studies considered) and/or its reporting? Why?

*This research was partly-funded by the National Institute for Health and Care Research Applied Research Collaboration North West Coast (NIHR ARC NWC). The views expressed are those of the authors and not necessarily those of the NHS, the NIHR, or the Department of Health and Social Care*.

## Key Points

1) Cardiorespiratory, respiratory and mixed training (especially when involving walking) can be safe for people following stroke who can walk on their own, and can improve key outcomes such as balance and mobility.
2) There is not enough evidence to support the effects on secondary outcomes linked to quality of life and mental health domains.
3) Further research would benefit from exploring the impact of cardiorespiratory, resistance, and mixed training dependent on time between stroke and intervention onset, stroke severity, and effects of the dose of intervention.
4) The involvement of patient groups to co-design appropriate and acceptable attention control in future studies, and the investigation of the cost-effectiveness of the interventions, are also recommended.
